# Meetings of Societies

**Published:** 1908-06

**Authors:** 


### MEETINGS OF SOCIETIES.

### Bristol Medico-Chirurgical Society.

*March 11th*, 1908.

Dr. Henry Waldo, President, in the Chair.

Dr. Carey Coombs showed specimens from the **myocardium in acute rheumatism,** drawing attention to the nodules composed of aggregations of large spindle-shaped, polynuclear cells occurring in the neighbourhood of arterioles. They were chiefly met with in the muscle of the left ventricle, near the apex. He was inclined to regard them as the result of a fibro-blastic reaction (peculiar, perhaps) to the rheumatic virus.

Mr. W. Roger Williams inquired whether there was any similarity between these formations and the sub-cutaneous nodules which Charcot had considered such a certain sign of the "arthritic diathesis."

Mr. S. V. Stock read notes of a case of **Carcinoma of the Breast,** in which metastasis occurred after an interval of twenty-four years. The breast had been amputated twenty-four years previously. A few days after the operation the dressings were accidentally set on fire, and the patient was severely burnt; erysipelas supervened, but after a prolonged illness a good recovery was made, and the patient was free from any recurrence. In May, 1907, the patient noticed a lump in the right iliac fossa, which Sir Thomas Smith (who had performed the first operation) and Mr. Bowlby diagnosed as a growth in the right ovary, but regarded as inoperable. The growth increased rapidly, nodules appeared above the clavicle, on the sternum, and ultimately in many other situations, either in glands or in subcutaneous tissues. Some glands were removed from the groin with a local anæsthetic, which under the microscope showed clear evidence of being carcinoma secondary to a primary mammary growth. Ultimately the patient died (eight months after first noticing the mass in the pelvis), with symptoms of cerebral metastasis. The reasons for the long period of quiescence and the possible channels of transmission were considered at some length.

Mr. Hey Groves pointed out the necessity of accepting with some reserve the pathological report that the carcinoma of the gland submitted to examination was indubitably secondary to that in the breast, and also the diagnosis of the mass in the pelvis as being ovarian, in the absence of proof obtained by operation or by necropsy. On the whole, however, he agreed that probably the explanation given was the correct one, although such an occurrence must be very rare.

Mr. Roger Williams drew attention to the relative chronicity of mammary cancer, saying that from data he had collected one case in twelve lasted for more than five years, several cases lasting fifteen, twenty, and even thirty years with ultimate recurrence; particularly was this true of melanotic sarcoma. He questioned the part played by lymphatic paths in recurrence or metastasis. Might not the traumatism of the operation liberate into the circulation cells in a pre-cancerous stage, which lodge, rest, and then owing to some change in the metabolism of the body take on their usual malignancy in some remote situation?

Mr. C. B. Goulden read a paper on **Lachrymal Obstruction,** in which, after briefly reviewing the anatomy and physiology of the lachrymal sac and nasal duct, he went on to deal with the causes of obstruction, most of them being due primarily to diseases in the nasal mucosa, or to caries of the nasal and lachrymal bones from tuberculosis or syphilis. He insisted on the dangers of acute or chronic dacryo-cystitis, especially the risk of operation on an eye where dacryo-cystitis was present. The treatment advocated for epiphora without mucocele consisted in bringing the puncta into correct apposition with the globe either by slitting the canaliculus, or, better, by producing with the actual cautery a scar between the inner canthus and punctum, which by contraction would restore the latter to its true position. For mucocele he advised treatment of the nose if required, and syringing of the sac, but unless this proved successful in six weeks, excision of the sac must be resorted to. For dacryo-cystitis the sac should invariably be excised. The use of probes and styles he utterly condemned.

Dr. Ogilvy was of the opinion that the presence of a mucocele did not actually justify so gloomy a view of operations on the eye. He had of necessity had to operate in the face of this complication, and his patients had not fared so badly as might have been expected, nor to his mind was probing and the use of styles entirely without value.

Mr. Cyril Walker pointed out that excision of the sac had been the universal rule until Bowman suggested and proved the efficacy of probing. Probably the advocates of excision would learn the same lessons by which Bowman had profited, and might admit that of two not wholly satisfactory procedures, Sir William had adopted the more generally useful. He drew attention to some methods by which the difficulties and dangers of passing probes and inserting styles could be minimised. If excision of the sac were practised, care should be taken to avoid dividing the tarsal ligament.

Dr. A. Fells read notes of a case of **Urobilinuria associated with Polycythæmia** occurring in a woman, aged 37, who had been long resident in India, and had suffered from sunstroke and many bouts of fever. In 1901 she returned to England, and first began to complain of shivering fits and attacks of pain in the left side, which radiated down the leg. In 1906 she became liable to attacks of fainting, with extreme irritability and restlessness. She then had intermittent pyuria with pyrexia. The right kidney was explored, and being found movable but otherwise healthy was fixed by suturing. Subsequently headache, neuralgia, insomnia, constipation and profound muscular weakness successively developed, and the urine became of a clear port-wine colour. The blood-count showed an increase of erythrocytes (6,500,000) without leucocytosis. Eventually the patient died with increasing myasthenia and paralysis extending gradually from the extremities to the respiratory centres. The urine had been examined, and reported to contain "urobilin." The case was probably one of auto-intoxication from the alimentary tract. The patient had taken sulphonal, but not to excess.

Dr. Symes considered that a case showing such persistent and long-continued urobilinuria was probably unique. He agreed with Dr. Fell's suggesiton of toxæmia as the explanation.

Dr. Edgeworth said he felt no doubt that the specimen of urine shown contained hæmatoporphyrin, and that the case was one of sulphonal poisoning, ending, as such cases frequently do, in peripheral neuritis.

Dr. Nixon inquired as to the tests which had been relied on for establishing the presence of urobilin and the absence of hæmatoporphyrin, since he was convinced that, whatever else the urine contained, the colour was due to hæmatoporphyrin.

Dr. Waldo detailed a case which he had treated of a man who had died of chronic sulphonal poisoning with hæmatoporphyrinuria. The similarity in the course of the disease to that of Dr. Fell's case was striking, and he could not help thinking that probably hæmatoporphyrin was present in this instance also.

A small sub-committee was asked to examine the urine, and report on its composition to the Society at some later date.

---

*April 8th,* 1908.

Dr. Henry Waldo, President, in the Chair.

Mr. W. Sampson Handley gave an address, illustrated with lantern slides, on **Breast Cancer in its relations to the Lymphatic System.**

Mr. Roger Williams alluded to the difficulty of explaining certain metastases by lymphatic permeation, and asked, for example, how lymphatic permeation could account for a secondary cancer in the suprarenal gland from primary cancer of the tongue, as in a recorded case? Secondary cancer in a uterine myoma from primary breast cancer could hardly be explained by permeation. How could permeation explain the fact that secondary cancer had been passed to a fœtus in utero? The speaker thought the permeation hypothesis was destined to have only temporary vogue. The address was discussed also by Messrs. Mole, Groves, Carwardine, Walters and Vickery, and Mr. Handley replied.—A cordial vote of thanks was given to Mr. Sampson Handley for his address.

---

*May 13th,* 1908.

Dr. Henry Waldo, President, in the Chair.

Dr. Shingleton Smith proposed and Mr. Nelson C. Dobson seconded the following Resolution, viz.: "That this Society welcomes the proposal to establish a University in Bristol, believing that it will act as a stimulus to learning, serve as a centre of research and prove a lasting benefit to education in the City, more especially to the Medical School, which has hitherto felt the handicap of its inability to confer degrees upon its Students." The Resolution was passed unanimously.

Dr. Watson Williams showed a case illustrating the **Osteo-plastic operation for Frontal Sinus Disease.** The patient, a man, had suffered from severe frontal and general headache, and had had a purulent nasal discharge. A left antral abscess pointing in the cheek had been first dealt with. Later, on the same side, the frontal sinus had been opened, and the eyebrow incision prolonged down the side of the nose. The lachrymal groove was then exposed through a second incision at the lower margin of the orbit, the nasal process of the maxilla, etc., sawn through from within and turned outwards as an osteo-plastic flap, and the ethmoid cells and sphenoidal sinus thoroughly curetted. Lastly the frontal sinus on the other side had been dealt with through another incision, and also the antrum on that side. The disfigurement was practically *nil* and the relief of symptoms great.

Dr. Michell Clarke showed (1) a specimen of **Tumour of the Pituitary Body,** and (2) gave an account of two cases of **Tumour of the Temporo-Sphenoidal Lobe of the Brain.**

Dr. Fortescue-Brickdale read a paper on **Calmette's Ophthalmo-Reaction.** (Page 112.)

J. Lacy Firth,
J. A. Nixon, *Hon. Sec.*

